# Unexpected phenotypic and molecular changes of combined glucocerebrosidase and acid sphingomyelinase deficiency

**DOI:** 10.1242/dmm.049954

**Published:** 2023-05-10

**Authors:** Marcus Keatinge, Matthew E. Gegg, Lisa Watson, Heather Mortiboys, Nan Li, Mark Dunning, Deepak Ailani, Hai Bui, Astrid van Rens, Dirk J. Lefeber, Anthony H. V. Schapira, Ryan B. MacDonald, Oliver Bandmann

**Affiliations:** ^1^ Bateson Centre, Firth Court, University of Sheffield, Western Bank, Sheffield S10 2TN, UK; ^2^ Sheffield Institute for Translational Neuroscience (SITraN), University of Sheffield, 385a Glossop Road, Sheffield S10 2HQ, UK; ^3^ Centre for Discovery Brain Sciences, Chancellor's Building, The University of Edinburgh, Edinburgh EH16 4TJ, UK; ^4^ Department of Clinical and Movement Neurosciences, UCL Queen Square Institute of Neurology, London NW3 2PF, UK; ^5^ Eli Lilly and Company, Drop Code 1940, Indianapolis, IN 46285, USA; ^6^ Department of Laboratory Medicine, Translational Metabolic Laboratory, Radboud University Medical Center, Nijmegen NL-6525 GA, The Netherlands; ^7^ Department of Neurology, Donders Institute for Brain, Cognition and Behaviour, Radboud University Medical Centre, Nijmegen NL-6525 GA, The Netherlands

**Keywords:** Parkinson's disease, Glucocerebrosidase 1, Acid sphingomyelinase, Zebrafish, Gene-gene interaction

## Abstract

Heterozygous variants in *GBA1*, encoding glucocerebrosidase (GCase), are the most common genetic risk factor for Parkinson's disease (PD). Moreover, sporadic PD patients also have a substantial reduction of GCase activity. Genetic variants of *SMPD1* are also overrepresented in PD cohorts, whereas a reduction of its encoded enzyme (acid sphingomyelinase or ASM) activity is linked to an earlier age of PD onset. Despite both converging on the ceramide pathway, how the combined deficiencies of both enzymes might interact to modulate PD has yet to be explored. Therefore, we created a double-knockout (DKO) zebrafish line for both *gba1* (or *gba*) and *smpd1* to test for an interaction *in vivo*, hypothesising an exacerbation of phenotypes in the DKO line compared to those for single mutants. Unexpectedly, DKO zebrafish maintained conventional swimming behaviour and had normalised neuronal gene expression signatures compared to those of single mutants. We further identified rescue of mitochondrial Complexes I and IV in DKO zebrafish. Despite having an unexpected rescue effect, our results confirm ASM as a modifier of GBA1 deficiency *in vivo.* Our study highlights the need for validating how genetic variants and enzymatic deficiencies may interact *in vivo*.

## Teaser

**Summary:** Simultaneous inhibition of *gba1* and *smpd1* gene function (both implicated in Parkinson's disease) unexpectedly rescues neuronal gene expression and mitochondrial defects, and extends the lifespan of *gba1* mutant zebrafish.

## INTRODUCTION

There is compelling evidence of an excessive burden of lysosomal disease gene variants and lysosomal dysfunction in Parkinson's disease (PD) (Robak et al., 2017; Wallings et al., 2019). Bi-allelic mutations in glucocerebrosidase 1 (*GBA1*; encoding glucocerebrosidase or GCase) cause Gaucher's disease, a lysosomal storage disorder (LSD), whereas heterozygous mutations are the most common and strongest genetic risk factor for sporadic PD, with a prevalence of ∼5-20% depending on the population investigated (Siebert et al., 2014; Orr-Urtreger et al., 2009; Neumann et al., 2009). PD patients also exhibit reduced GCase activity in different tissues, including the brain (Gegg et al., 2012), regardless of their *GBA1* mutation status (Gegg et al., 2012; Parnetti et al., 2017; Atashrazm et al., 2018).

**Fig. 1.**
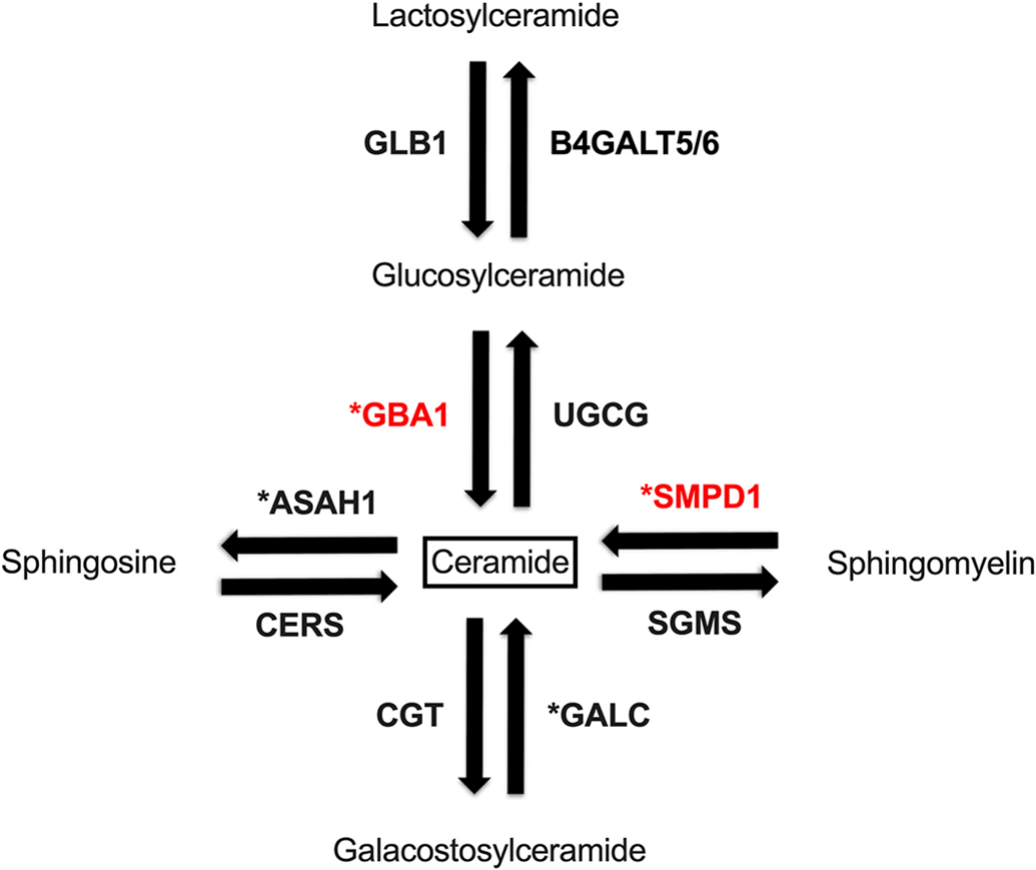
**Representative image of the ceramide pathway.** The enzymes involved in the ceramide pathway under study (GBA1 and SMPD1) are highlighted in red; enzymes marked with asterisks are linked to PD.

In a similar fashion to *GBA1* mutations, homozygous mutations in *SMPD1* (encoding acid sphingomyelinase or ASM) also cause an LSD, in this case, Niemann–Pick disease, whereas heterozygous *SMPD1* variants are associated with increased risk of sporadic PD (Alcalay et al., 2019; Gan-Or et al., 2013; Foo et al., 2013; Mao et al., 2017; Dagan et al., 2015). Owing to their rarity within the PD population, the functional significance of these *SMPD1* variants is still not completely understood (Alcalay et al., 2019; Usenko et al., 2022). However, a reduction of ASM activity is correlated with an earlier age of disease onset in PD as well as in other synucleinopathies, including dementia with Lewy bodies and multiple system atrophy (Alcalay et al., 2019; Usenko et al., 2022). Both *GBA1* and *SMPD1* encode lysosomal enzymes that converge on ceramide metabolism (Fig. 1). Therefore, an additive interaction between these two enzymes is biologically plausible but awaits experimental confirmation. We hypothesised that ASM deficiency could worsen the functional consequences of GCase deficiency, aggravating phenotypes that could potentially lead to, or enhance, neurodegeneration.

Zebrafish (*Danio rerio*) are an attractive vertebrate model to study the biological effect of both monogenic PD genes and genetic risk factors for PD (Flinn et al., 2013; Larbalestier et al., 2022), including *gba1* deficiency (Keatinge et al., 2015). We had previously characterised a *gba1* mutant zebrafish line (*gba1^−/−^*) (Keatinge et al., 2015) and demonstrated its usefulness to study gene-gene interactions (Watson et al., 2019). *gba1^−/−^* zebrafish faithfully model key features of GCase deficiency or Gaucher's disease, including Gaucher cell accumulation, marked inflammation with microglial infiltration, mitochondrial dysfunction and neurodegeneration (Keatinge et al., 2015). *gba1^−/−^* larvae develop normally but *gba1^−/−^* juvenile zebrafish then rapidly deteriorate from 10-12 weeks onwards and die between 12 and 14 weeks.

As expected, combined GCase and ASM deficiency acted synergistically on key sphingolipid metabolites in the *gba1^−/−^*;*smpd1^−/−^* double-mutant zebrafish. However, instead of a worsening of phenotypes, we unexpectedly observed markedly prolonged survival and conventional swimming behaviour in *gba1^−/−^*;*smpd1^−/−^* mutants compared to the behaviour of the *gba1^−/−^* (single) mutant zebrafish. RNA sequencing (RNAseq)-based pathway analysis confirmed the restoration of neuronal health in *gba1^−/−^;smpd1^−/−^* mutants compared to that in the *gba1^−/−^* mutants. Mechanistic experiments identified a rescue effect of combined GCase and ASM deficiency on the function of mitochondrial Complexes I and IV in *gba1^−/−^*;*smpd1^−/−^* mutants compared to the marked but distinct mitochondrial dysfunction in *gba1^−/−^* or *smpd1^−/−^* single mutant zebrafish. The mitochondrial rescue led to an abrogation of oxidative membrane damage, further reflecting the overall restorative effect of ASM deficiency on neuronal health in GCase deficiency. Our study highlights the need of functional, mechanistic validation for the interaction of any putative genetic/enzymatic risk factors for human diseases in suitable model systems, rather than readily assuming an additive effect.

## RESULTS

### *smpd1^−/−^* zebrafish display abolished ASM activity and marked sphingolipid accumulation

**Fig. 2.**
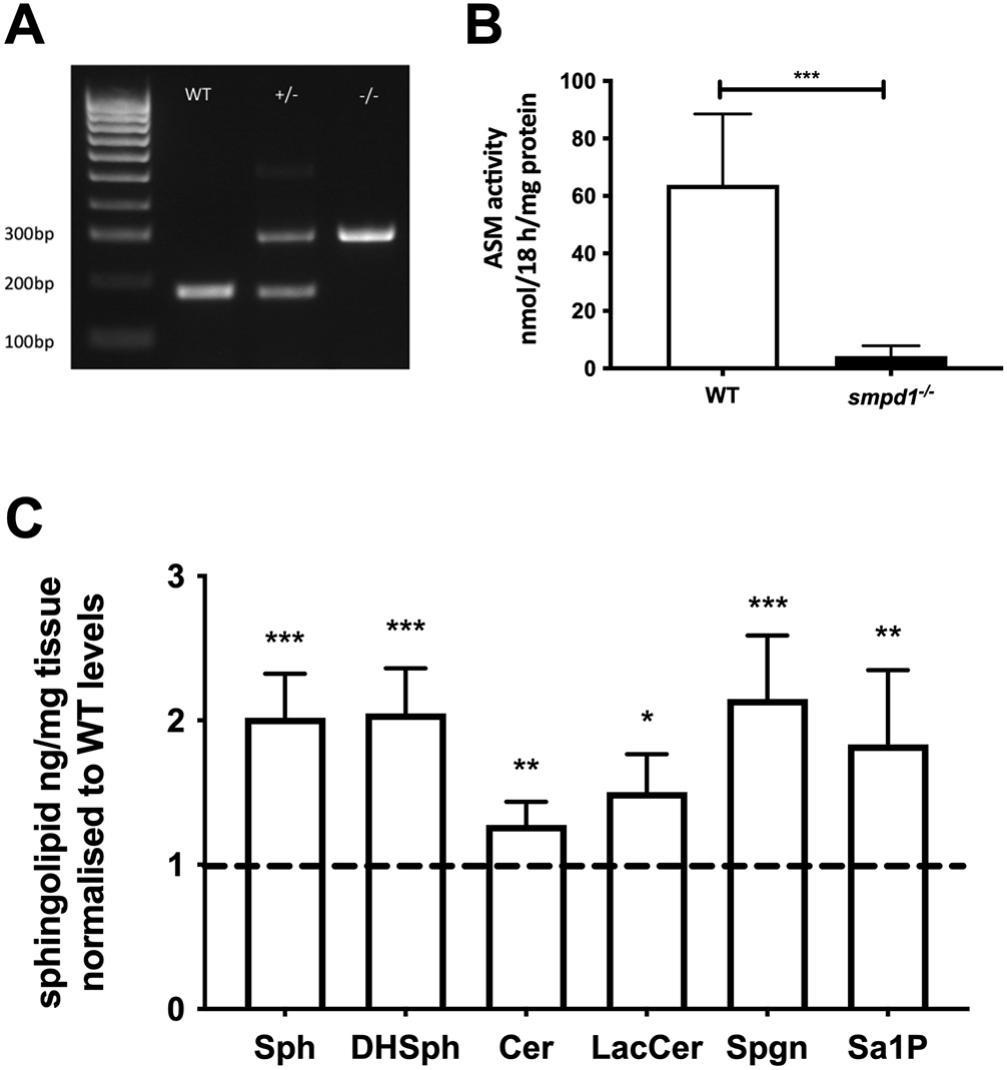
**Genetic and biochemical characterisation of the *smpd1^−/−^* mutant zebrafish line.** (A) Representative genotyping gel of the *smpd1^−/−^* alleles (5 bp deletion and 136 bp insertion) demonstrating WT, *smpd1^+/−^* and *smpd1^−/−^*. (B) Acid sphingomyelinase (ASM) enzymatic activity in *smpd1^−/−^* compared to WT controls (*n*=6 larvae at 5 dpf per genotype, *P*=0.006 by two-tailed unpaired Welch's *t*-test). (C) Quantification of sphingolipid metabolites, namely, sphingomyelin (Sph), dihydro-sphingomyelin (DHSph), ceramide (Cer), lactosylceramide (LacCer), sphinganine (Spgn) and sphinganine 1 phosphate (Sa1P). All metabolites shown are the C18 neuronal species. Data represented are the mean±s.d. *P<0.05; **P<0.01; ***P<0.001 (two-tailed unpaired *t*-test).

We initially hypothesised that deficiency of both GCase and ASM enzymes may synergise, leading to a further aggravation of *gba1*-deficient phenotypes *in vivo*. To address this, we identified a single *smpd1* orthologue in zebrafish (ENSDARG00000076121) with 59% shared identity to the human *SMPD1* gene at both the DNA and the protein level. CRISPR/Cas9 technology was used to generate a stable *smpd1* mutant line (*smpd1^−/−^*)*.* The selected mutant allele contained a 5 bp deletion and 136 bp insertion within exon 3, resulting in a frame shift and the generation of a premature stop codon (Fig. 2A; Fig. S1). Enzymatic activity of ASM in *smpd1^−/−^* at 5 days post fertilisation (dpf) was reduced by 93% (*P*=0.006, Fig. 2B). The large reduction in ASM enzymatic activity resulted in a significant increase of key glycolipid substrates in the *smpd1^−/−^* larvae already at 5 dpf (Fig. 2C). The *smpd1^+/−^* line was crossed with the *gba1^+/−^* line to generate *gba1^+/−^;smpd1^+/−^*. The latter were subsequently in-crossed to generate double mutants, single mutants and wild-type (WT) controls for all subsequent experiments. At each in-cross, larvae were genotyped at 3 dpf and then raised in genotype-specific tanks. Every genotype was present in its expected Mendelian ratio (1/16) during genotyping at the larval stages (Table S1), but only the WT, *gba1^−/−^*, *smpd1^−/−^* and *gba1^−/−^;smpd1^−/−^* lines were raised for experiments.

### Combined ASM and GCase deficiency synergistically increases sphingolipid metabolites

**Fig. 3.**
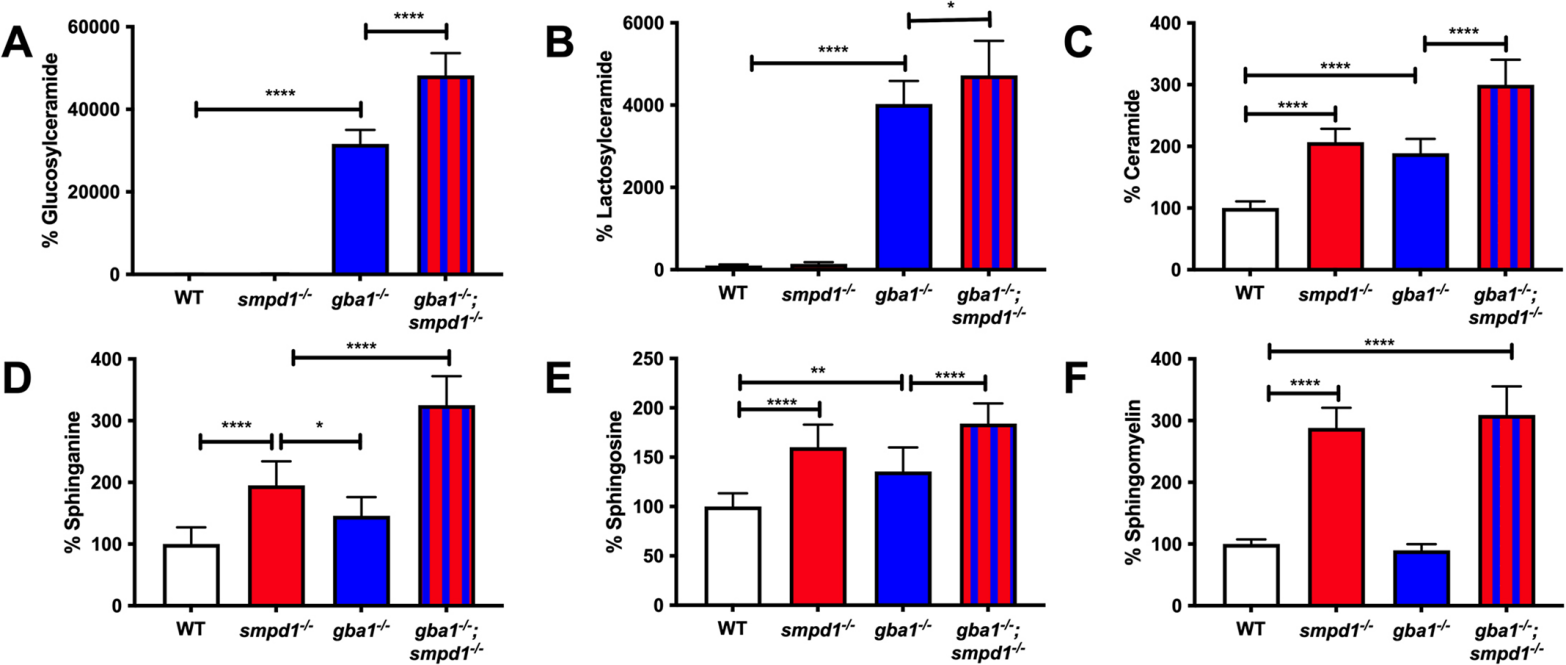
**Accumulation of key glycolipids across *gba1^−/−^* and *smpd1^−/−^* single-mutant and *gba1^−/−^;smpd1^−/−^* double-mutant genotypes.** Relative glucosylceramide (A), lactosylceramide (B), ceramide (C), sphinganine (D), sphingosine (E) and sphingomyelin (F) levels in WT, single-mutant *gba1^−/−^* and *smpd1^−/−^*, and double-mutant *gba1^−/−^;smpd1^−/−^* zebrafish. *n*=10 brains from 12-week-old zebrafish used per group. Data represented are the mean±s.d. *P<0.05; **P<0.01; ****P<0.0001 (two-way ANOVA with Tukey's multiple comparisons).

We had previously reported marked sphingolipid accumulation in *gba1^−/−^* zebrafish (Keatinge et al., 2015). We hypothesised that combined (enzymatic) GCase and ASM deficiency would synergistically increase distinct sphingolipid subtypes. Using mass spectrometry, a comprehensive panel of glycolipid substrates was analysed in the brains of *gba1^−/−^* and *smpd1^−/−^* single mutant as well as in *gba1^−/−^;smpd1^−/−^* double mutant zebrafish and WT controls at 12 weeks of age. As expected, a marked additive effect of combined GCase and ASM deficiency was observed for glucosylceramide levels (the direct substrate of GCase) (Fig. 3A). This was likely due to both a block in glucosylceramide catabolism and metabolic compensation in the flux of sphingolipid generation. Combined GCase and ASM deficiency also resulted in an additive effect on lactosylceramide, ceramide and sphinganine levels (Fig. 3B-D). Sphingosine levels were increased in *gba1^−/−^;smpd1^−/−^* compared to those in WT, reflecting an increase compared to those in *gba1^−/−^* but not compared to those in *smpd1^−/−^* (Fig. 3E). Unexpectedly, there was no synergistic effect in sphingomyelin levels in the *gba1^−/−^;smpd1^−/−^* double mutants (Fig. 3F).

**Fig. 4.**
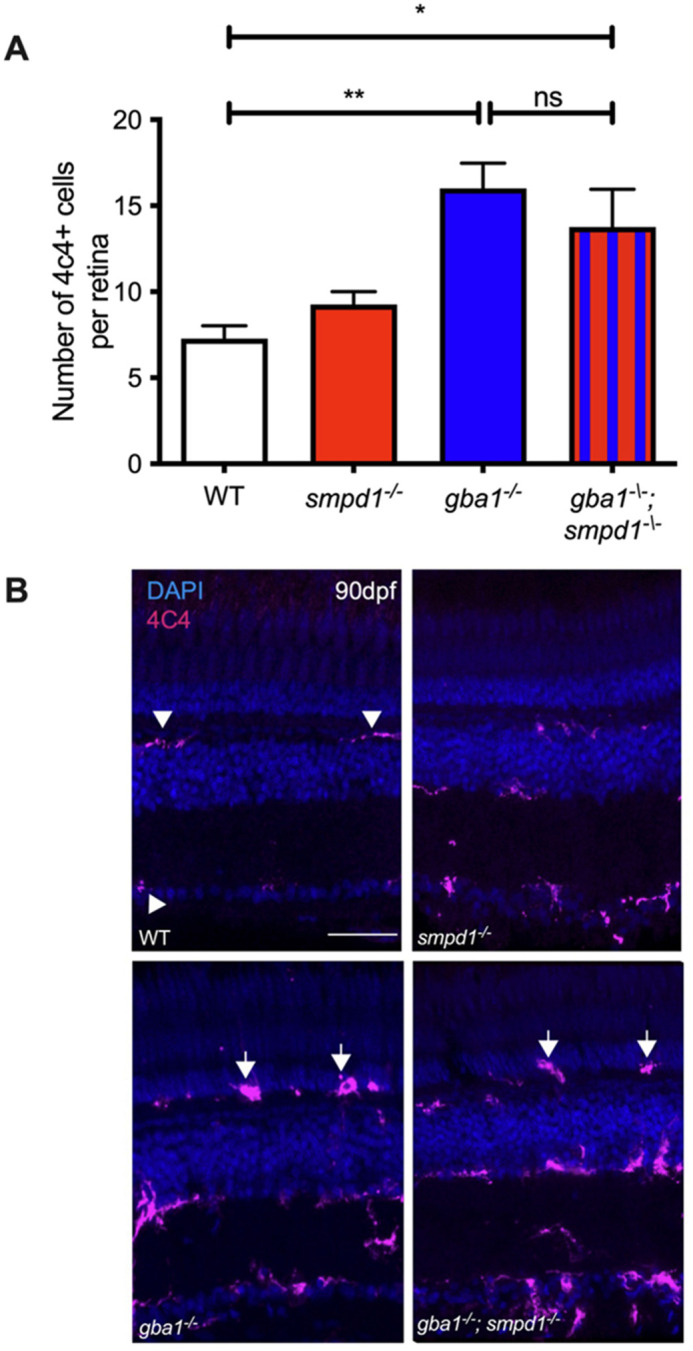
**Gaucher cell accumulation in the retina of *gba1^−/−^;smpd1^−/−^* double mutants.** (A) Analysis of the numbers of 4C4-positive cells within the retina revealed comparatively significant increases of ∼50% in both *gba1^−/−^* (*P*=0.0026) and *gba1^−/−^;smpd1^−/−^* mutants (*P*=0.0193) compared to those in WT, despite enhancement of key glycolipid levels in the double mutants. 4C4-positive cell counts in *smpd1^−/−^* were not statistically different to those in WT. Data represented are the mean±s.d. *P<0.05; **P<0.01 (two-way ANOVA). *n*= 4 for all groups. (B) 4C4-positive cells in the retina of the *gba1^−/−^* and *gba1^−/−^;smpd1^−/−^* double mutants were localised throughout the retina and appeared larger and rounder (arrows) compared to ramified WT 4C4-positive cells (arrowheads). Scale bar: 50 μm.

The inflammation markers chitotriosidase (CHIT1) and β-hexosaminidase (heterodimer or homodimer of HEXA and/or HEXB) are markedly increased in the serum of Gaucher's disease patients and used as biomarkers to monitor disease activity (Grabowski, 2012). We previously observed a marked increase in chitotriosidase and β-hexosaminidase activity in *gba1^−/−^* zebrafish brain tissue at 12 weeks (Keatinge et al., 2015). As key GCase substrates were synergistically increased in *gba1^−/−^;smpd1^−/−^* double-mutant zebrafish, we investigated whether combined GCase and ASM inactivation may also result in a further increase of chitotriosidase and β-hexosaminidase activity. Unexpectedly, *gba1^−/−^;smpd1^−/−^* double-mutant zebrafish displayed a similar increase in chitotriosidase and β-hexosaminidase activity compared to that seen for *gba1^−/−^* zebrafish (Fig. S2). Furthermore, we had previously detected Gaucher cell invasion in the central nervous system of end-stage *gba1^−/−^* zebrafish (Keatinge et al., 2015). Analyses of the retinas across all genotypes demonstrated similar Gaucher cell invasion in the double mutants (Fig. 4), with both *gba1^−/−^* and *gba1^−/−^;smpd1^−/−^* mutants showing an ∼50% increase in cells positive for 4C4 (which marks microglia) compared to WT. These 4C4-positive cells in both *gba1^−/−^* and *gba1^−/−^;smpd1^−/−^* mutants also tended to be larger and rounder, indicative of the Gaucher cells we had previously described. These data suggest persistent yet unaltered neuroinflammatory states in the double mutants despite a marked synergistic increase in sphingolipid metabolites.

### ASM deficiency unexpectedly prolongs survival in GCase deficiency

**Fig. 5.**
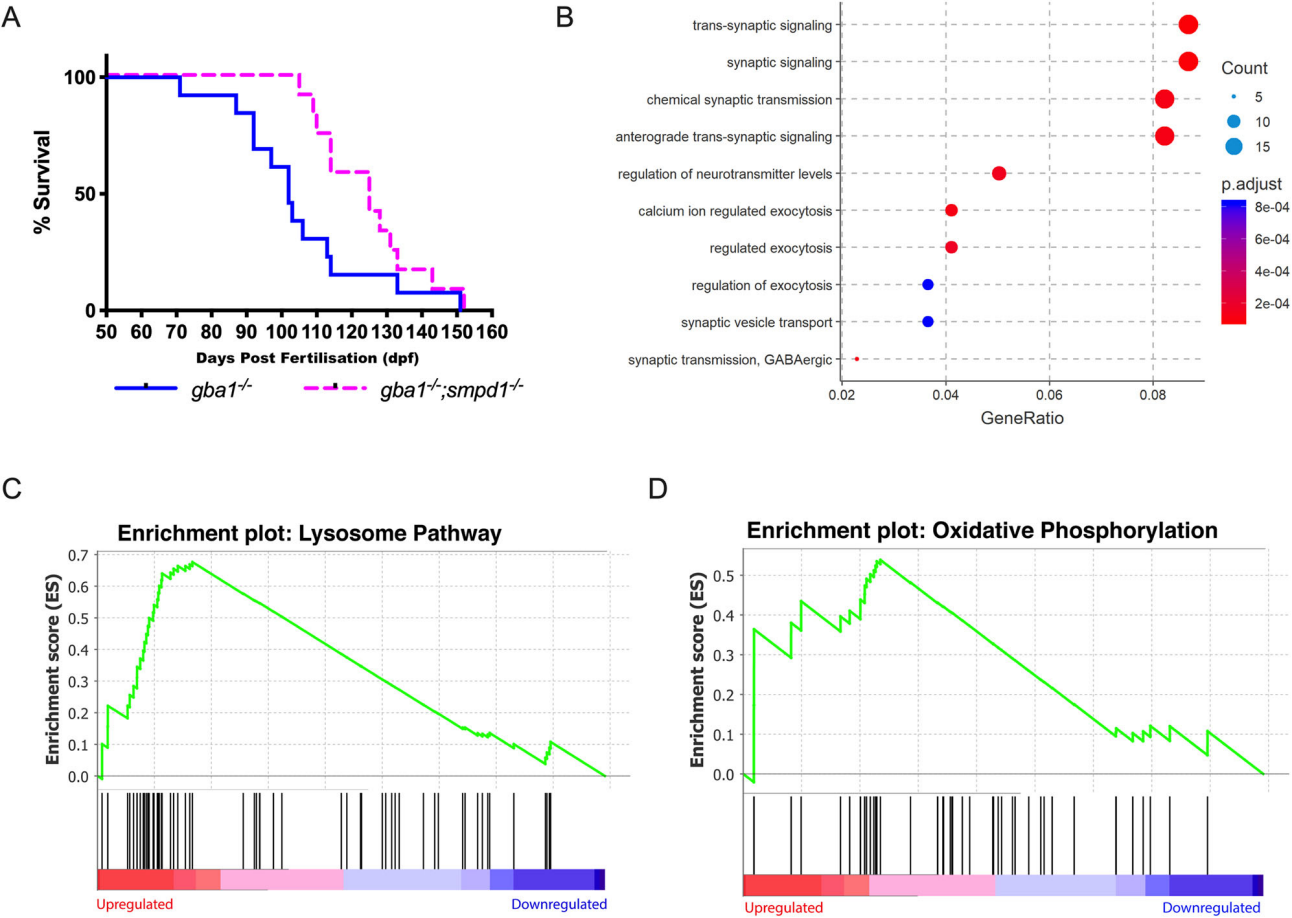
**ASM deficiency improves survival and rescues neuronal dysfunction in *gba1^−/−^* zebrafish.** (A) Disease-free survival analysis of *gba1^−/−^;smpd1^−/−^* double-mutant (*n*=13) compared to *gba1^−/−^* single-mutant (*n*=12) zebrafish. *P*=0.0055 by Gehan–Breslow–Wilcoxon test. Animals were culled for humane reasons when they could no longer consistently maintain buoyancy. (B) The Comparative GO analysis indicates marked global impairment of neuronal function in *gba1^−/−^* but restoration of neuronal health in *gba1^−/−^;smpd1^−/−^* **.** A differential expression analysis was first used to identify genes with statistically significant difference between *gba1^−/−^* and *gba1^−/−^;smpd1^−/−^*. Genes with adjusted *P*-value<0.05 and log_2_(fold change) >1 or <−1 were used to identify enriched biological processes amongst genes that were over- and under-expressed in *gba1*^−/−^. ClusterProfiler identified significantly enriched GO terms within the gene expression changes, which were plotted by GeneRatio (ratio of the differentially expressed genes with one particular GO term to the total number of differentially expressed genes). The lists of over- and under-expressed genes were analysed separately. Shown are the ten GO terms with highest gene ratios amongst under-expressed genes, all relating to key aspects of normal neuronal function and homeostasis. The pathways downregulated in *gba1^−/−^* compared to WT were normalised in the double mutants. Each GO term is coloured according to the adjusted *P*-value and ranked according to gene ratio. The size of the point is then scaled according to the number of differentially expressed genes with the GO term. (C,D) Lysosomal pathway genes and oxidative phosphorylation pathway genes are upregulated in *gba1^−/−^* but normalised in *gba1^−/−^;smpd1^−/−^*. The comparison of RNAseq-based transcription levels in the respective pathways between *gba1^−/−^* and *gba1^−/−^;smpd1^−/−^* revealed that both lysosomal pathway genes (C) and oxidative phosphorylation pathway genes (D) were enriched with marked upregulation of both pathways in *gba1^−/−^* compared to WT and *gba1^−/−^;smpd1^−/−^*. The *x*-axis ranks all differentially expressed genes based on the rank metric score from the most upregulated (left) to the most downregulated (right) for either pathway. The vertical black lines show the location of pathway genes in the entire ranked list from the *gba1^−/−^* expression changes, compared to WT and double mutants. The *y*-axis is the degree to which a set of pathway genes is overrepresented at the extremes (up- or downregulated) of the entire ranked list of all differentially expressed genes within the genome. A peak in enrichment score (green line) demonstrates an enrichment of pathway genes amongst all over- or under-represented genes. A sharp peak demonstrates how highly upregulated each pathway is within the *gba1^−/−^* group compared to WT and double mutants.

The marked additive effect of combined GCase and ASM deficiency on sphingolipid levels led us to hypothesise that ASM deficiency would further worsen the motor phenotype and shorten survival in *gba1^−/−^;smpd1^−/−^* double-mutant zebrafish. Unexpectedly, genetic inactivation of ASM led to a complete rescue of this behaviour in the *gba1^−/−^;smpd1^−/−^* double-mutant zebrafish [Movie 1 (WT), Movie 2 (*smpd1^−/−^*), Movie 3 (*gba1^−/−^*) and Movie 4 (*gba1^−/−^;smpd1^−/−^*)]. Importantly, disease-free survival, in which animals could consistently maintain buoyancy, was also markedly increased by 22% in *gba1^−/−^;smpd1^−/−^* double-mutant zebrafish compared to that in *gba1^−/−^* zebrafish (median survival of 102 dpf in *gba1^−/−^* and 125  dpf in *gba1^−/−^;smpd1^−/−^*, *P*=0.0055; Fig. 5A). Despite not exhibiting the same barrel rolling phenotype as the *gba1^−/−^* mutants, and also being able to maintain their buoyancy, the *gba1^−/−^;smpd1^−/−^* double mutants would ultimately be found unresponsive at the bottom of the tank and were thus culled for humane reasons. We also raised *smpd1^−/−^* mutants to determine their lifespan, but never encountered a decrease in viability compared to that of WT zebrafish, even up to the age of 18 months (data not shown).

### RNAseq-based pathway analysis confirms restored neuronal health in *gba1^−/−^;smpd1^−/−^* zebrafish

We next applied RNAseq-based pathway analysis to further elucidate the underlying mechanisms of the observed rescue effect. The differential gene expression analysis in all four genotypes (WT, *gba1^−/−^* and *smpd1^−/−^* single mutants, and *gba1^−/−^;smpd1^−/−^* double mutants) identified a total of 512 genes that were dysregulated in *gba1^−/−^* but rescued in *gba1^−/−^;smpd1^−/−^*. Amongst these, 219 genes were downregulated and 293 genes were upregulated in *gba1^−/−^* compared to wild-type and *gba1^−/−^;smpd1^−/−^* [adjusted *P*-value≤0.05, |log_2_(fold change)|≥1]. We next employed ClusterProfiler analysis on Gene Ontology (GO) categories to identify functionally relevant pathways within the rescued gene sets. Key neuronal pathways including the GO terms for synaptic signalling, chemical synaptic transmission and calcium ion-regulated exocytosis were markedly downregulated in *gba1^−/−^* but normalised in *gba1^−/−^;smpd1^−/−^* (Fig. 5B). This suggests that key aspects of neuronal function were restored in the *gba1^−/−^;smpd1^−/−^* double mutants.

We also observed an enrichment of upregulated genes in *gba1^−/−^*compared to those in *gba1^−/−^;smpd1^−/−^* in a broad range of GO terms, the top five of which are thought to regulate muscle function. However, as our RNAseq analysis was carried out on brain tissue, we consider these changes to be of limited relevance (Fig. S3). Upregulation of the inflammatory signature in *gba1^−/−^* was retained in *gba1^−/−^;smpd1^−/−^* but not further enhanced (data not shown).

As both GCase and ASM are lysosomal hydrolases, we specifically focused on the effect of isolated GCase deficiency in *gba1^−/−^* compared to the effect of combined GCase and ASM deficiency in *gba1^−/−^;smpd1^−/−^* on lysosomal transcriptomic pathways. Gene set enrichment analysis led to the identification of 27 leading-edge, dysregulated lysosomal genes, which account for the enrichment signal of the pathway. The expression of these 27 lysosomal genes was increased in *gba1^−/−^* compared to wild-type and *gba1^−/−^;smpd1^−/−^* (Fig. 5C; Table S2). Amongst these 27 genes, acid hydrolases contributed the most. *Cathepsin L.1* (*ctsl.1*), involved in the initiation of protein degradation, ranked as the top-rescued gene. The apparent normalisation of lysosomal gene expression profiles in *gba1^−/−^;smpd1^−/−^* was in contrast to the observed marked increase in a wide range of sphingolipid levels in *gba1^−/−^;smpd1^−/−^* compared to the sphingolipid levels in *gba1^−/−^ or smpd1^−/−^* single mutants (see above).

We had previously observed marked mitochondrial dysfunction in *gba1^−/−^*. We therefore also focussed on the analysis of mitochondrial genes involved in the oxidative phosphorylation pathway. This leading-edge mitochondrial gene subset included 16 genes encoding the subunits of the Complexes I, II, IV and V in the mitochondrial electron transport chain (Table S3). Interestingly, gene set enrichment analysis showed an upregulation of this mitochondrial gene subset in *gba1^−/−^*, presumably as a compensatory mechanism to the impaired function of the mitochondrial respiratory chain, but showed similar mitochondrial gene expression levels in WT and *gba1^−/−^;smpd1^−/−^* (Fig. 5D).

### Restoration of mitochondrial Complex I and IV function in *gba1^−/−^;smpd1^−/−^*

**Fig. 6.**
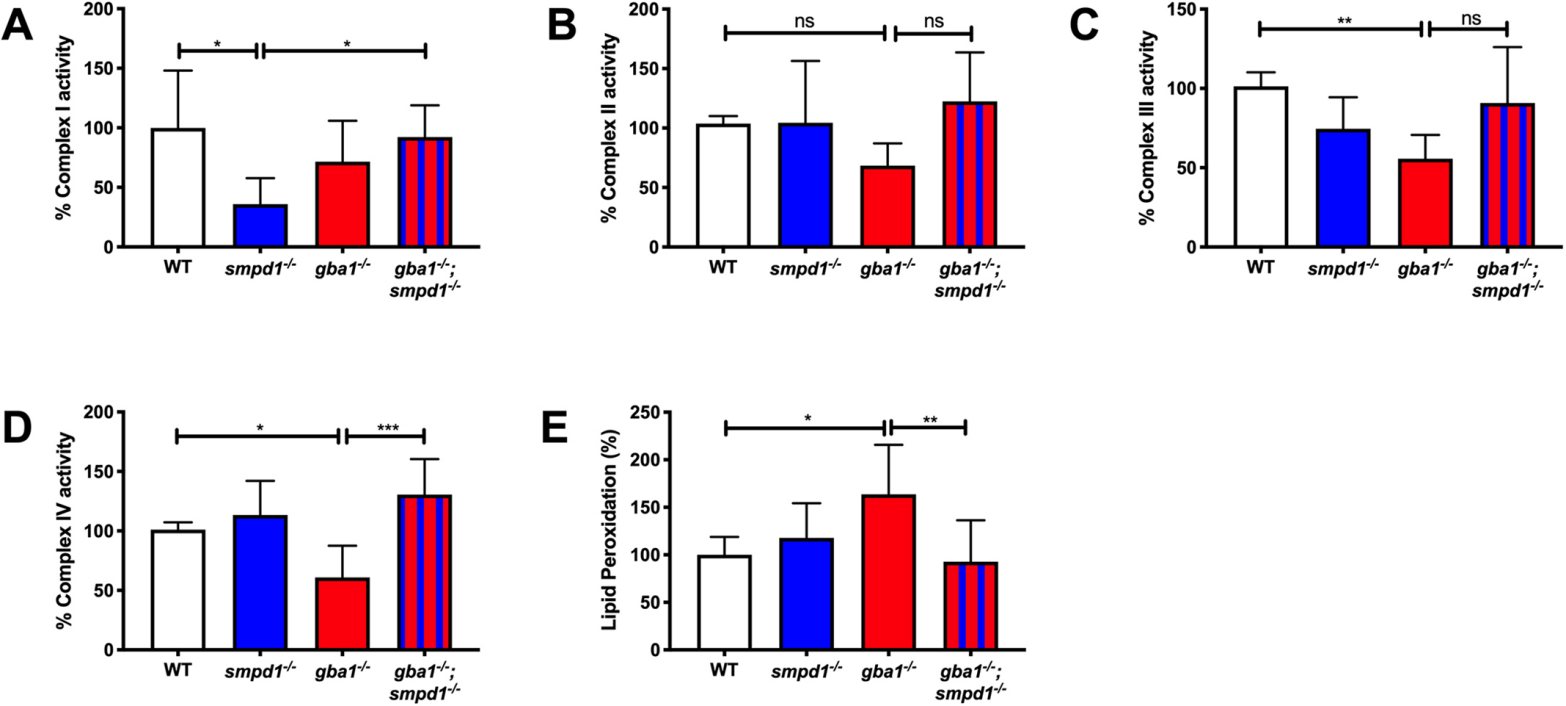
**Mitochondrial respiratory chain function and lipid peroxidation.** (A) Complex I activity was reduced in *smpd1^−/−^* by 64±34.77% (mean±s.d.) compared to that in WT (*P*=0.0198). Complex I activity was normalised in *gba1^−/−^;smpd1^−/−^* with an increase of 56±21.9% compared to that in *smpd1^−/−^* (*P*=0.0445). (B) Complex II activity was similar across the different genotypes (*P*>0.05). (C) Complex III activity was reduced in *gba1^−/−^* compared to that in WT by 45±14.99% (*P*=0.0091) and increased by 35±35.2% in *gba1^−/−^;smpd1^−/−^* compared to that in *gba1^−/−^*, but this did not reach significance (*P*>0.05). (D) Complex IV activity was reduced in *gba1^−/−^* by 40±26.79% compared to that in WT (*P*=0.0491), but completely rescued in *gba1^−/−^;smpd1^−/−^* double mutants with an increase of 69±26.79% compared to that in *gba1^−/−^* (*P*=0.0005). For all mitochondrial complex activity measurements, six brains were used for each genotype. (E) Mitochondrial lipid peroxidation levels were increased by 63±51% in *gba1^−/−^* compared to those in WT (*P*=0.0214), but reduced by 71±43.48% compared to those in *gba1^−/−^* and thus effectively normalised to WT levels in *gba1^−/−^;smpd1^−/−^* double mutants (*P*=0.0094). For lipid peroxidation experiments, *n*=6-8 zebrafish bodies were used for each genotype. Significance in both mitochondrial respiratory chain assays and analyses of lipid peroxidation levels was determined by two-way ANOVA with Tukey's multiple comparison test using 12-week-old brain material. Data represented are the mean±s.d. ns, not significant; *P<0.05; **P<0.01; ***P<0.001.

We next compared the mitochondrial respiratory chain function across all four genotypes to further determine whether the normalised gene expression levels for oxidative phosphorylation-related genes would be reflected in normalised mitochondrial function. Complex I activity was reduced by 65% in *smpd1^−/−^* compared to WT levels (*P*=0.0198, Fig. 6A) but restored to 92% of WT levels in *gba1^−/−^;smpd1^−/−^* (*P*=0.0445, Fig. 6A). Complex II activity was not significantly altered in any of the genotypes (Fig. 6B). Complex III activity in *gba1^−/−^* was reduced by 45% compared to WT levels (*P*=0.0091, Fig. 6C) as previously observed (Keatinge et al., 2015). Complex III activity in the *gba1^−/−^;smpd1^−/−^* double-mutant zebrafish was reduced by only 9% compared to WT levels; however, this did not reach significance compared to the levels observed in *gba1^−/−^* (*P*=0.1688). Complex IV activity was unchanged in *smpd1^−/−^* compared to WT but reduced by 40% in *gba1^−/−^* compared to WT, as previously reported (*P*=0.0491, Fig. 6D). Remarkably, there was a marked improvement of complex IV activity in *gba1^−/−^;smpd1^−/−^* with an increase in activity of 69% compared to that in *gba1^−/−^* (*P*= 0.0005, Fig. 6D). Thus, there was rescue of mitochondrial respiratory chain function by mitochondrial Complexes I and IV, in which ASM deficiency normalised mitochondrial Complex IV function in *gba1^−/−^* and GCase deficiency normalised mitochondrial Complex I function in *smpd1^−/−^*. Malfunction of the mitochondrial respiratory chain can result in oxidative stress and subsequent lipid peroxidation. We therefore investigated whether the observed rescue in the activity of mitochondrial Complexes I and IV resulted in reduced oxidative stress-related damage. Mitochondrial lipid peroxidation was increased in whole *gba1^−/−^* adult fish by 63% above WT levels (*P*= 0.0214, Fig. 6E). As predicted, lipid peroxidation levels were reduced by 70% in *gba1^−/−^;smpd1^−/−^* double mutants compared to *gba1^−/−^* and thus effectively normalised (*P*=0.0094, Fig. 6E).

## DISCUSSION

Biochemically, GCase and ASM both play a key role in sphingolipid metabolism (Hannun and Obeid, 2008; Quinville et al., 2021). Unexpectedly, we observed a rescue of motor behaviour and a marked prolongation of life expectancy following combined GCase and ASM deficiency, despite clear evidence of an additive effect on the intracellular level of key sphingolipids and their metabolites. The remarkable rescue effect of mitochondrial Complexes I and IV in *gba1^−/−^;smpd1^−/−^* on behaviour and survival suggests a central role of mitochondrial dysfunction in GCase deficiency. The profound normalisation of neuronal function in *gba1^−/−^;smpd1^−/−^*, as indicated in our RNAseq-based pathway analysis, is in keeping with the observation of the rescued motor phenotype. The normalisation of intracellular homeostasis is also reflected by the normalisation of both lysosomal and mitochondrial transcriptional pathways.

Mitochondrial dysfunction is a key feature of both familial and sporadic PD, as well as LSDs, as the mitochondrial network and lysosomal system are also known to be tightly interlinked (Kim et al., 2021; Magalhaes et al., 2018; Plotegher et al., 2019; Plotegher and Duchen, 2017a,b; Raimundo et al., 2016). ASM activity must also be tightly controlled, as either too much or too little has been shown to negatively affect mitochondrial function, depending on the cell type, tissue or experimental paradigm under study (Niu et al., 2022; Gillmore et al., 2022; Novgorodov et al., 2019). However, when focussed on neuronal health, ASM inhibition consistently ameliorates phenotypes in the context of neuronal loss (Novgorodov et al., 2019; Lee et al., 2014; Hagemann et al., 2020). A plausible rescue mechanism of mitochondrial function in the double-knockout (DKO) zebrafish could be glutamate/calcium signalling. Neuronal GCase deficiency *in vitro* has been shown to sensitise mitochondria to physiological levels of glutamate (Plotegher et al., 2019). This leads to pathological responses in calcium signalling and downstream mitochondrial dysfunction (Plotegher et al., 2019). Conversely, ASM deficiency *in vitro* has been shown to cause a decreased vulnerability to glutamate-linked excitotoxicity in neurons (Yu et al., 2000). This was not only linked to a decrease in intracellular calcium levels, but also to a decrease in oxidative stress (Yu et al., 2000). Inhibiting ASM function in primary oligodendrocyte culture can also rescue glutamate-induced mitochondrial dysfunction (Novgorodov et al., 2019). Of note, nine genes involved in calcium ion-regulated exocytosis were downregulated in *gba1^−/−^* single mutants but subsequently normalised in the DKO mutant; namely, *syt2a*, *syt7a*, *cplx3a*, *cadpsa*, *snap47*, *cacna1hb*, *rims1b*, *cbarpb* and *napbb*.

An alternative mechanism could be intracellular redistribution of the sphingolipid profile in double mutants. We only detected a synergistic increase in sphingolipid levels in the DKO mutant compared to the *gba1^−/−^* single mutant, but not a normalisation. However, this does not exclude an effect on subcellular localisation of a specific sphingolipid metabolite that may underpin the observed rescue mechanism. Sphingolipid signalling is vitally important for many varied intercellular and intracellular processes (Hannun and Obeid, 2008). Sphingolipid signalling must remain highly compartmentalised due to its pleiotropic effects (Canals and Clarke, 2022; Ivanova, 2020; Piccinini et al., 2010). However, due to technical reasons, we used bulk brain tissue for our metabolite analysis, which would therefore not allow for the detection intercellular and intracellular sphingolipid differences. Future work should involve metabolic analyses of sphingolipids separated by cell type and by specific cellular fractions to produce a spatial understanding of the distinct sphingolipid metabolism and distribution across the different *gba1^−/−^* and *smpd1^−/−^* genotypes. Furthermore, whole-body analyses using recently developed techniques to monitor *in situ* glucosylceramide generation would give novel insights into the glycolipid dysregulation in our double mutants (Katzy et al., 2022).

Intriguingly, deficiency of another LSD gene, *asah1b*, which functions on a separate arm of the ceramide pathway to *smpd1* (Fig. 1), also ameliorates *gba1-*deficient phenotypes *in vivo* and *in vitro*. Biallelic *ASAH1* mutations cause the LSD Farber disease in humans. By developing a DKO zebrafish for *gba1^−/−^* and *asah1b^−/−^*, Lelieveld et al. (2022) demonstrated that *asah1b* deficiency also led to a rescue of behavioural and neuronal phenotypes in a similar manner to that of our DKO *gba1^−/−^*;*smpd1^−/−^* zebrafish. In keeping with our own data, the rescue effect observed was not due to an amelioration of neuroinflammation, as DKO zebrafish retained the upregulation of *tnfb*, *il1b* and *apoeb* exhibited by *gba1^−/−^* (Lelieveld et al., 2022). Similarly, Kim et al. (2018) demonstrated that pharmacological inhibition of ASAH1 led to a significant reduction in *GBA1-*linked cellular phenotypes, including accumulation of ubiquitinated proteins and α-synuclein in dopaminergic neuronal cultures derived from PD *GBA1^+/−^* patients.

For practical reasons, we modelled combined enzymatic deficiency using homozygous, and not heterozygous, mutants for *gba1* and *smpd1*, intrinsically modelling LSDs and not PD. However, the unexpected nature of our results demonstrates the need for future characterisation of combined partial LSD gene deficiencies in the wider context of PD.

## MATERIALS AND METHODS

### Zebrafish husbandry

All larval and adult zebrafish were housed at the University of Sheffield; experimental procedures were in accordance with the UK Home Office Animals (Scientific Procedures) Act 1986 (project license PPL 70/8437, held by O.B.). Adult zebrafish were housed at a density of 20 per tank, on a cycle of 14 h of light, 10 h of dark. Adults and embryos were kept at a constant temperature of 28°C.

### Mutant line generation and line maintenance

The *gba1^−/−^* mutant lines was generated using TALEN technology (Keatinge et al., 2015). The *smpd1^−/−^* mutant line was generated by the CRISPR/Cas9 method as previously described (Keatinge et al., 2021; Hruscha et al., 2013). The following ultramer template was used: 5′-AAAGCACCGACTCGGTGCCACTTTTTCAAGTTGATAACGGACTAGCCTTATTTTAACTTGCTATTTCTAGCTCTAAAACGGATTGAGGCTTGTGTCTCCCTATAGTGAGTCGTATTACGC-3′. The *smpd1^−/−^* line was genotyped using the following primers: F, 5′-AGCCGTGGTGGTTTCTACAG-3′, and R, 5′-CCTTCTCTCCCTTGTTCTCG-3′. The *smpd1^−/−^* line was crossed to *gba1^+/−^* to generate double-heterozygous individuals. These were subsequently in-crossed to generate double mutants, single mutants and WT controls. At each in-cross, larvae were genotyped at 3 dpf by larval tail biopsy as previously described (Wilkinson et al., 2013). Each genotype was raised in genotype-specific tanks at a density of 10-15 fish per tank. All individuals were re-genotyped at 10 weeks post fertilisation. For survival curves, animals were culled for humane reasons when they could no longer maintain consistent buoyancy.

### Immunohistochemistry

WT and mutant fish were fixed in 4% paraformaldehyde overnight at 4°C before removal of the eye, which was incubated in 30% sucrose in PBS overnight at 4°C. Eyes were then embedded in OCT compound (Tissue Tek O.C.T.; Sakura, 4583) and cryosectioned at 20 µm (Leica). Slides were rehydrated in PBS before blocking for 1 h at room temperature using 150 µl blocking solution (1% sheep serum, 5% bovine serum albumin, 0.3% Triton X-100 and 0.1% Tween-20 in PBS). Slides were then incubated with 150 µl primary antibody solution (4C4; a generous gift from Noemie Hamilton, Department of Biology, University of York, York, UK; 1:50 dilution in block solution; antibody registry ID: AB_10013752) overnight at 4°C. After incubation, slides were washed three times in PBS for 20 min followed by incubation with secondary antibody (Alexa Fluor 647 anti-mouse IgG; Invitrogen, A-21235) for 2 h. Slides were then washed in PBS three times for 20 min, before adding Fluoroshield with DAPI (Sigma-Aldrich, F6057-20ML) and applying a glass coverslip. Slides were imaged on a Zeiss LSM 900 confocal microscope using a 40× water-immersion objective.

### Biochemical activity assays and mass spectrometry

ASM activity was determined using homogenates prepared as follows: tubes containing 20 embryos (5 dpf) were sonicated in 500 μl MilliQ water and centrifuged (3400 ***g***). Then, 20 μl of supernatant was incubated with the substrate HMU-PC (6-hexadecanoylamino-4-methylumbelliferyl-phosphorylcholine; 0.66 mM, Moscerdam Substrates, The Netherlands) at pH 5.2 and 37°C for 2 h. Fluorescence intensity was measured at 415 nm (excitation) and 460 nm (emission) using a plate reader (Perkin Elmer, LS55). Lysosomal and mitochondrial enzyme assays as well as mass spectrometry were undertaken as previously described (Keatinge et al., 2015). Enzyme assays were performed on brain homogenates at 12 weeks post fertilisation at a concentration of 1 mg/ml and at 28°C. Chitotriosidase, β-galactosidase and β-hexosaminidase activity was measured using 4-methylumbelliferyl-β-N,N′,N″-triacetyl-chitotriose (Sigma-Aldrich), 4-methylumbelliferyl-galactopyranoside (Sigma-Aldrich) and methylumbelliferyl-2-acetamido2-deoxy-β-gluco-pyranoside (Sigma-Aldrich), respectively, all dissolved in the respective McIlvaine citrate–phosphate buffer.

### Lipid peroxidation assay

We were unable to isolate sufficient mitochondria from brain tissue to robustly measure lipid peroxidation signals above background levels. However, sufficient mitochondria could be isolated from 3-month-old adult zebrafish bodies to perform the assay robustly. Bodies were homogenised in ice-cold mitochondrial isolation buffer [ice-cold sucrose buffer (0.4 M phosphate buffer pH 7.4, 0.25 M sucrose, 0.15 M KCl, 40 mM KF and 1 mM N-acetyl-cysteine)]. The Abcam lipid peroxidation kit (ab118970) fluorometric assay was used to measure lipid peroxidation according to the manufacturer's instructions. Results were normalised to WT samples.

### RNA preparation for gene expression analysis

RNA was prepared from brain tissue of 12-week-old zebrafish. A TRIzol-based protocol was used to extract RNA from the tissue. Briefly, individual brains were homogenised in 250 µl TRI Reagent (Sigma-Aldrich) and incubated at room temperature before adding 50 µl chloroform (Thermo Fisher Scientific). The samples were centrifuged at 13,300 ***g*** and the top aqueous phase was collected and transferred to a separate tube. RNA was precipitated from the aqueous phase by mixing with an equal volume of isopropanol (Thermo Fisher Scientific) and centrifugation at 13,300 ***g***. The precipitated RNA was resuspended in DEPC-treated water (Thermo Fisher Scientific), and its concentration and quality were quantified using the Nanodrop 1000 Spectrophotometer. Approximately 750 ng of high-quality total RNA, with an RNA integrity number of 9 or above, was used in the preparation of sequencing libraries using the NEB Ultra II Directional RNA Library Prep Kit (New England Biolabs, E7760), following the polyA mRNA workflow [NEBNext® Poly(A) mRNA Magnetic Isolation Module]. Libraries were individually indexed and pooled for sequencing. Single-end 100 bp sequencing was performed on the Illumina HiSeq 2500 platform using Rapid Run mode with V2 chemistry.

### RNAseq analysis

Raw sequencing reads were processed using the bcbio workflow system. The quality of the samples was checked using FastQC and multiqc (Ewels et al., 2016). The salmon tool (v0.9.01) was used to quantify genes from the zebrafish reference transcriptome (Danio_rerio.GRCz11.98.gtf from https://www.ensembl.org/info/data/ftp/index.html) (Patro et al., 2017). The salmon files were then imported into R using the tximport Bioconductor package (Soneson et al., 2016). Unsupervised clustering and principal component analysis with DESeq2 revealed a batch effect corresponding to sample preparation date (Love et al., 2014). Differential expression was performed using DESeq2 incorporating a batch factor into the model. The contrast tested was between *gba1^−/−^*, double mutants and WT; a positive log_2_(fold change) indicated higher expression in *gba1^−/−^* single mutants. The ClusterProfiler Bioconductor package was used to identify enriched pathways in upregulated [adjusted *P*-value less than 0.05 and log_2_(fold change)>1] and downregulated genes [adjusted *P*-value less than 0.05 and log_2_(fold change)<−1] (Yu et al., 2012).

### Gene set enrichment analysis

The analysis was performed with Gene Set Enrichment Analysis (GSEA) software version 4.0.3. GSEA preranked analysis was used with default settings except for ‘Collapse/Remap to gene symbols’ set to ‘No_Collapse’. A ranked list used for the analysis was calculated with each gene assigned a score based on the adjusted *P*-value and the log_2_(fold change). Zebrafish lysosomal and mitochondrial gene sets were prepared by identifying zebrafish homologues of the genes in human gene sets in Molecular Signatures Database (MSigDB) v7.1.

### Statistical analysis

GraphPad Prism v6 software was used for statistical analysis and all error bars shown denote the mean±s.d. All experiments were performed in biological triplicate unless otherwise stated. All data were analysed with either two-tailed unpaired *t*-test or two-way ANOVA. Significance in all enzyme activity assays was determined by two-way ANOVA with Tukey's multiple comparison test.

## Supplementary Material

10.1242/dmm.049954_sup1 Supplementary information
