# Supplementary material for: Unexpected phenotypic and molecular changes of combined glucocerebrosidase and acid sphingomyelinase deficiency
Source: Dis Model Mech. 2023 May 10;16(6):dmm049954. doi: 10.1242/dmm.049954 (PMC10184672; doi:10.1242/dmm.049954)
Supplement: Supplementary information [file dmm-16-049954-s1.pdf]

smpd1 exon 3

CCGTGGTGGTTTCTACAGCGTGGAGGTTGAGCCTGGATTTGAAATAAAAAAATAATGCTAAAATTTAAATTTAAAT
AAAAATACAACATACAATACAAAATATATTTAAACCACAGAAAAACACTACAATTAAGCCACACATTAAACAAGTAA
AAACGCAGCNNTTTTTTTGTTTGAGGCTTGTGTCTCTGAACATGAACTTTTGCTCCAGAGAAAACTACTGGCTGA
TGGTGAACTCCACTGACCCAGCAGATCAGCTACAGTGGCTCATACAAATCCTGCAGGAGTCCGAGAACAAGGG
AGAGAAG

Fig. S1. CRISPR/Cas9 generated *smpd1* allele.

Using CRISPR/Cas9, we isolated a mutant allele in exon 3 containing a 5bp deletion (red script) with a 136bp insertion (blue script) leading to a frame shift and generation of a premature stop codon at codon 426 (full length WT smpd1: 676 amino acids).

Disease Models & Mechanisms • Supplementary information

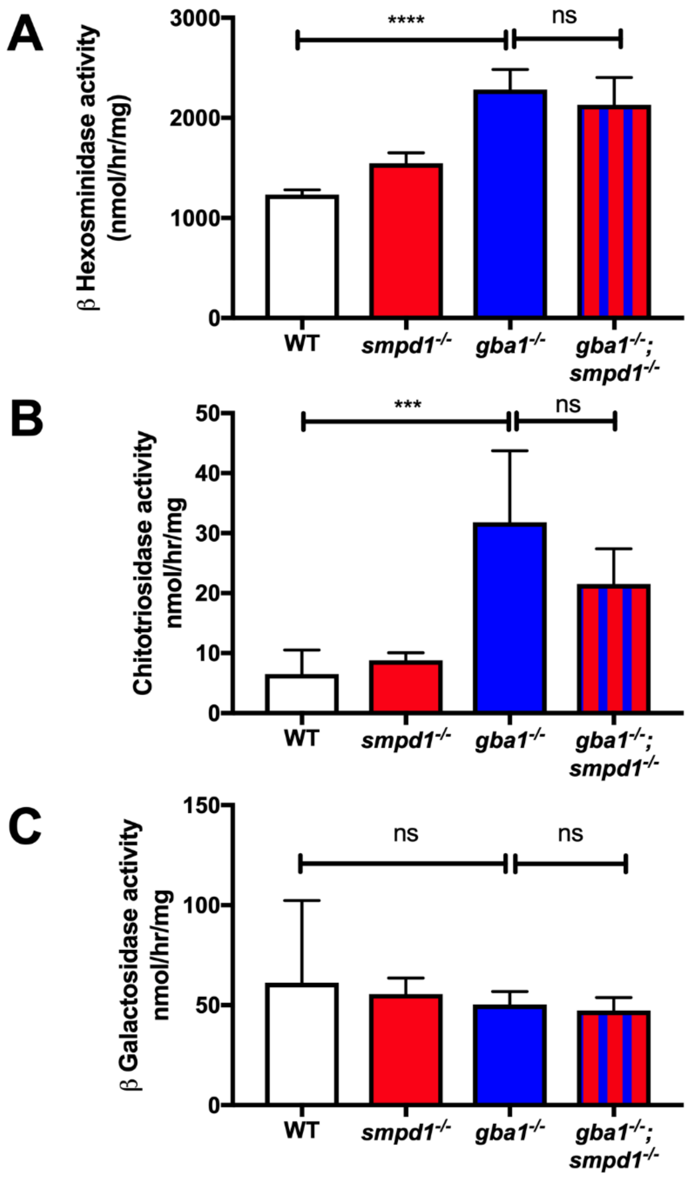

Fig. S2. GD biomarker activation is not substantially altered in *gba1$^{-/-}$;smpd1$^{-/-}$*.
(A) Hexosaminidase activity was not significantly changed between *smpd1$^{-/-}$* and WT, but markedly elevated by 85% in *gba1$^{-/-}$* (2284±200 nmol/hr/mg protein) compared to WT (1234±47.3nmol/hr/mg protein; p<0.0001). *gba1$^{-/-}$;smpd1$^{-/-}$* displayed a similar increase in activity of 70% above WT levels (2131±272.2nmol/hr/mg protein, p<0.0001) compared to *gba1$^{-/-}$*. *(B)* Chitotriosidase activity was not significantly changed in *smpd1$^{-/-}$* compared to WT, but increased in *gba1$^{-/-}$* by 389% (31.8±11.95 nm/hr/mg protein, p=0.0002) compared to WT (6.5±4.0 nm/hr/mh protein) and by 230% in *gba1$^{-/-}$;smpd1$^{-/-}$*showed (21.5±5.1nm/hr/mg protein, p=0.0177) compared to WT. *(C)* Beta galactosidase activity in all genotypes showed comparable activities of approximately 50nm/hr/mg protein. Significance in all enzyme activities assays was determined by two way ANOVA with Tukey's multiple comparison test with an n of 5 per group using 12 week brain material. Data represented are the mean ±SD. ****p<0.0001, ***p<0.001, **p<0.01 and *p<0.05.

Disease Models & Mechanisms • Supplementary information

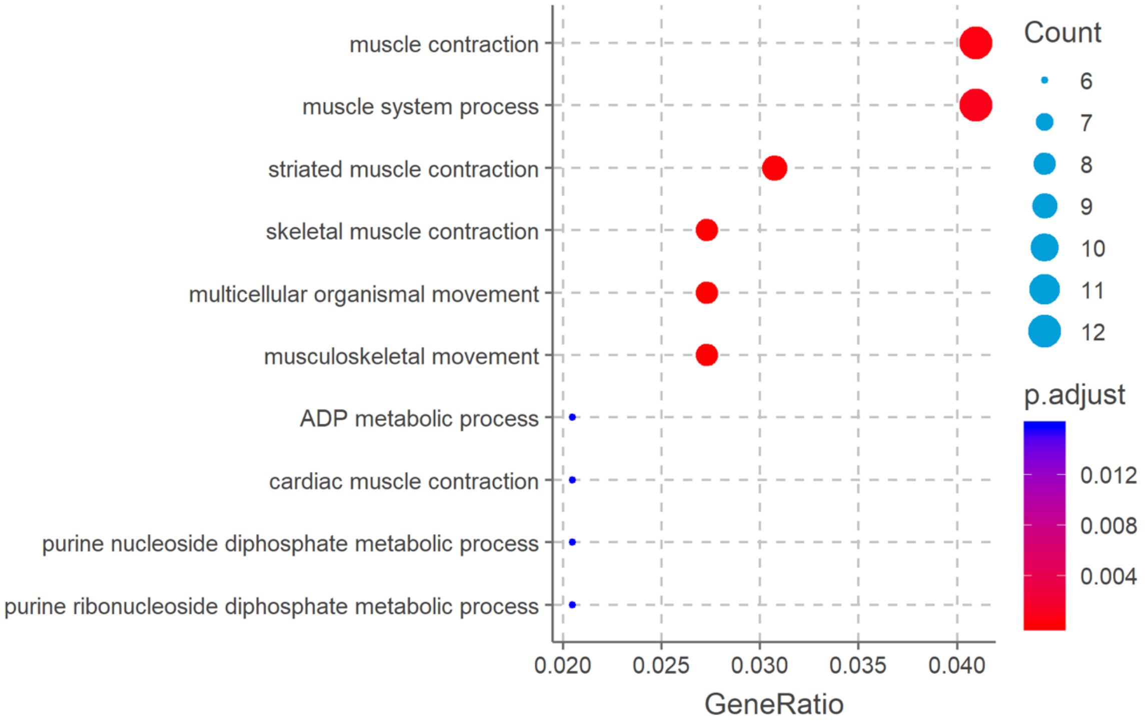

Fig. S3. Over-represented GO terms in the up-regulated genes. The top 10 GO terms as identified by ClusterProfiler from the list of statistically-significant genes (adjusted p-value < 0.05 and log2 fold-change > 1 or < -1 respectively. Each point is coloured according to the adjusted p-value for the GO term being over-represented, and the size of the point is scaled according to the number of differentially-expressed genes in the GO term. GO terms are ordered on the y-axis according to the size of the GO-term and adjusted p-value in the case of GO terms with the same number of genes.

Disease Models & Mechanisms • Supplementary information

Table S1. Observed and expected genotypes from *gba1*$^{+/-}$;*smpd1*$^{+/-}$ incrosses from 4 pooled clutches.

Genotype	WT	gba1+/-; WT	gba1-/-; WT	WT; smpd1+/-	gba1+/-; smpd1+/-	gba1-/-; smpd1+/-	WT; smpd1-/-	gba1+/-; smpd1-/-	gba1-/-; smpd1-/-	Total
n	14	48	15	26	48	27	23	35	15	251
Observed %	5.58%	19.12%	5.98%	10.36%	19.12%	10.76%	9.16%	13.94%	5.98%	100%
Expected %	6.25%	12.50%	6.25%	12.50%	25%	12.50%	6.25%	12.50%	6.25%	100%

Table S2. The leading-edge gene subset in lysosome pathway.

Symbol	Gene Name	Rank Metric Score
ctsl.1	cathepsin L.1	8.510105133
cd63	CD63 molecule	5.135530472
atp6ap1b	ATPase H+ transporting accessory protein 1b	5.114915371
lipf	lipase, gastric	3.06033802
fuca1.2	alpha-L-fucosidase 1, tandem duplicate 2	2.934919357
ctsba	cathepsin Ba	2.764246225
sftpbb	surfactant protein Bb	2.624561548
hexa	hexosaminidase A (alpha polypeptide)	2.622437954
zgc:110239	zgc:110239	2.503591537
acp5a	acid phosphatase 5a, tartrate resistant	2.363205671
gm2a	GM2 ganglioside activator	2.312668324
ctsa	cathepsin A	2.239866734
glb1	galactosidase, beta 1	2.198205709
dnase2b	deoxyribonuclease II beta	2.142905235
napsa	napsin A aspartic peptidase	1.98163259
atp6v0a1a	ATPase H+ transporting V0 subunit ca	1.948094964
ctsh	cathepsin H	1.844756842
manba	mannosidase, beta A, lysosoma	1.817932725
galns	galactosamine (N-acetyl)-6-sulfatase	1.797118545
dnase2	deoxyribonuclease II, lysosomal	1.73265183
npc1	Niemann-Pick disease, type C1	1.719917774
gnptg	N-acetylglucosamine-1-phosphate transferase subunit gamma	1.489329815
man2b1	mannosidase, alpha, class 2B, member 1	1.414416075
ap3b1a	adaptor related protein complex 3 subunit beta 1a	1.329013348
pla2g15	phospholipase A2, group XV	1.1423738
ppt1	palmitoyl-protein thioesterase 1 (ceroid-lipofuscinosis, neuronal 1, infantile)	1.051521778
atp6v0ca	ATPase H+ transporting V0 subunit ca	0.99467504

Disease Models & Mechanisms • Supplementary information

Table S3. The leading-edge gene subset in oxidative phosphorylation pathway.

Symbol	Gene Name	Rank Metric Score
atp6ap1b	ATPase H+ transporting accessory protein 1b	5.114915371
cox6a2	cytochrome c oxidase subunit 6A2	5.065165043
atp5pb	ATP synthase peripheral stalk-membrane	2.323343754
atp6v0a1a	ATPase H+ transporting V0 subunit a1a	1.948094964
atp6v0ca	ATPase H+ transporting V0 subunit ca	0.99467504
ndufb10	NADH:ubiquinone oxidoreductase subunit B10	0.839254677
ndufb6	NADH:ubiquinone oxidoreductase subunit B	0.643859327
ndufa11	NADH:ubiquinone oxidoreductase subunit A11	0.643721879
ndufv2	NADH:ubiquinone oxidoreductase subunit V2	0.574424922
cox4i1	cytochrome c oxidase subunit 4I1	0.573632479
sdhc	succinate dehydrogenase complex, subunit C, integral membrane protein	0.542427838
atp6v1g1	ATPase H+ transporting V1 subunit G1	0.497611314
cox4i2	cytochrome c oxidase subunit 4I2	0.428459734
cox5aa	cytochrome c oxidase subunit 5Aa	0.396667153
ndufa6	NADH:ubiquinone oxidoreductase subunit A6	0.356948555
ndufc2	NADH:ubiquinone oxidoreductase subunit C2	0.27688098

All videos were taken when the respective zebrafish were 12 weeks of age.

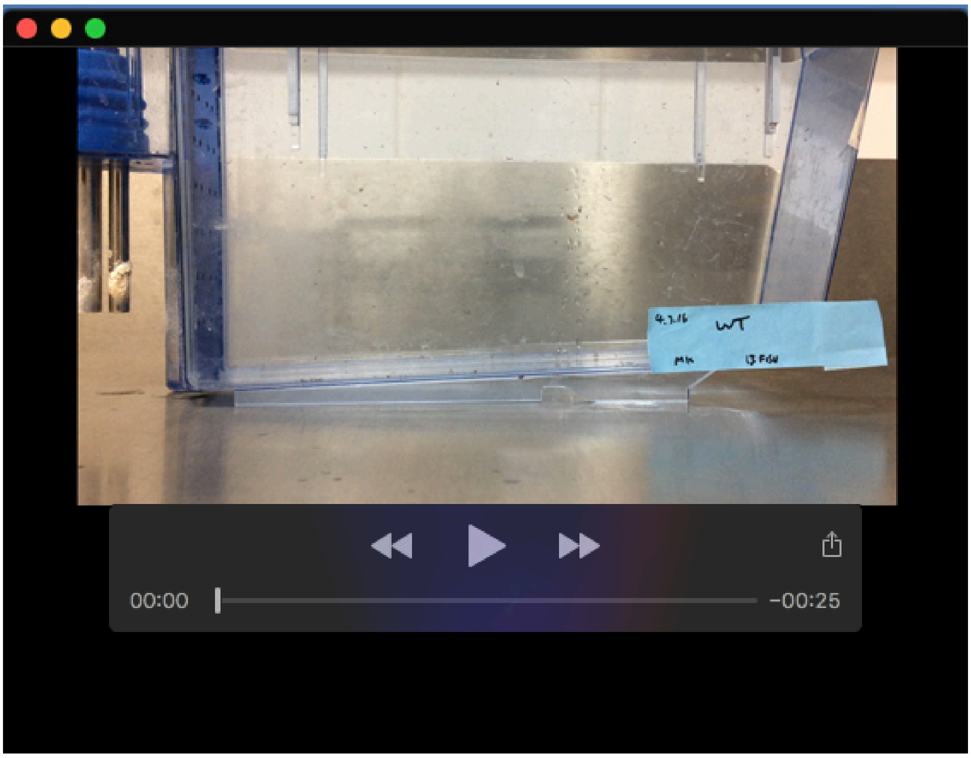

Movie 1. Swimming behaviour in WT. All adults placed into a novel tank swim to the bottom momentarily before resuming standard swimming, balance and buoyancy maintained.

Disease Models & Mechanisms • Supplementary information

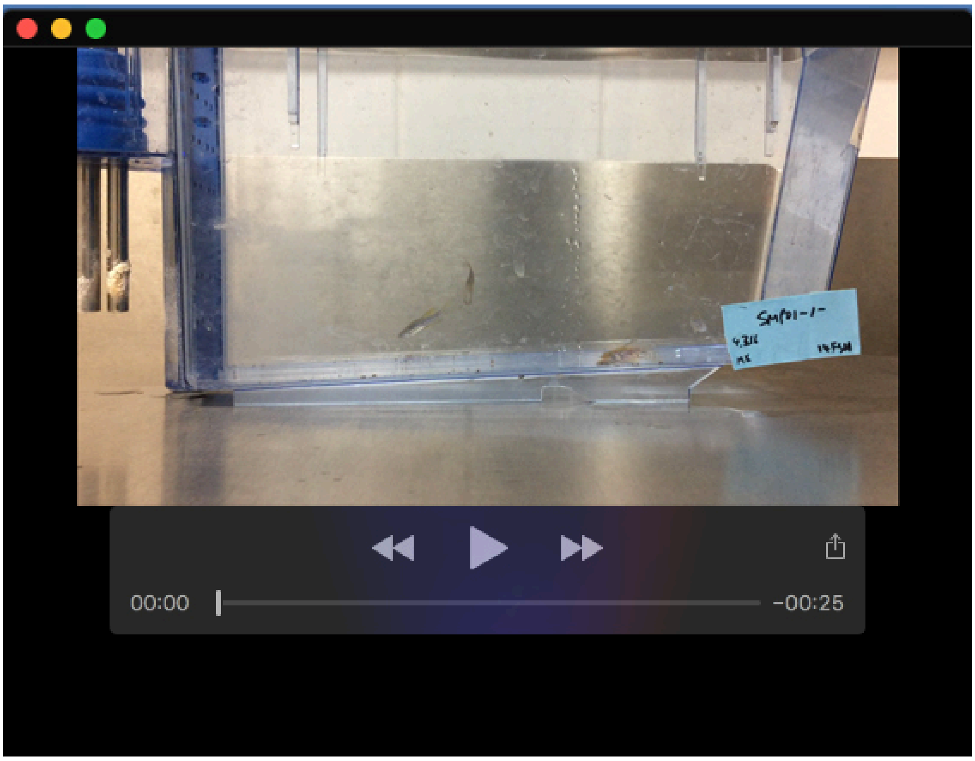

Movie 2. Swimming behaviour in *smpd*$^{-/-}$. All adults placed into a novel tank swim to the bottom momentarily before resuming standard swimming, balance and buoyancy maintained.

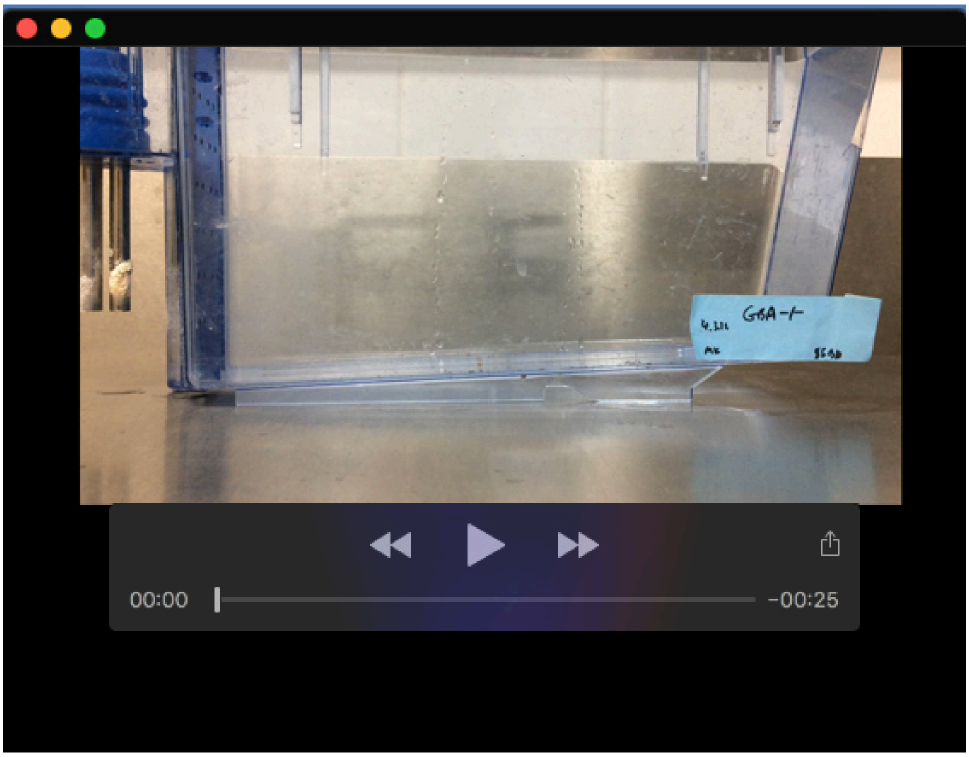

Movie 3. Markedly abnormal swimming behaviour in *gba*$^{-/-}$ with typical "corkscrew" swimming pattern. All adults placed into a novel tank swim in circular motions with balance and buoyancy defects. These increase with frequency and duration at end stage until they need to be culled for humane reasons.

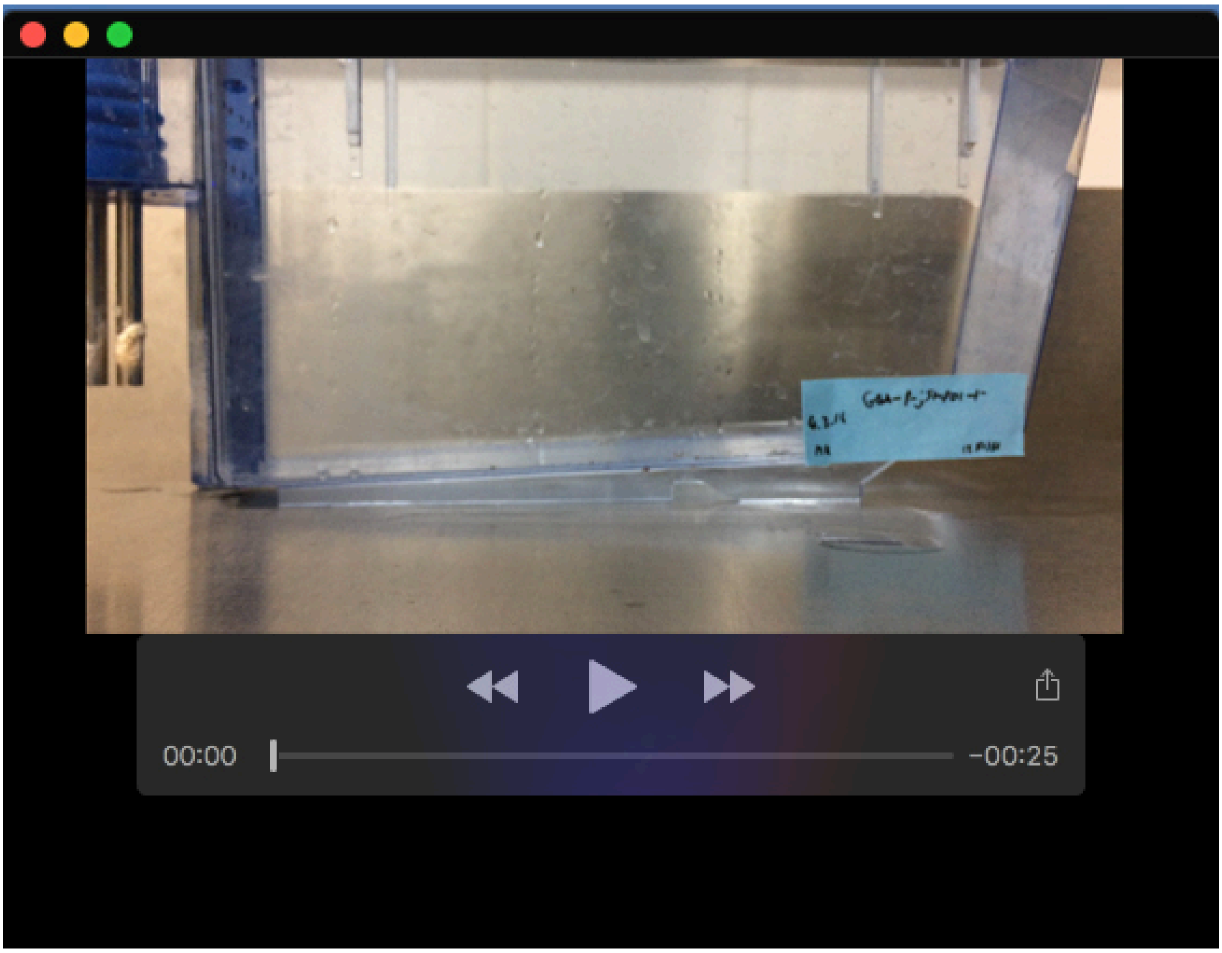

Movie 4. Swimming behaviour in *gba*$^{-/-}$;*smpd*$^{-/-}$. All adults placed into a novel tank swim to the bottom momentarily before resuming standard swimming, balance and buoyancy maintained.
